# High-Sensitivity Troponin T and Soluble Form of AXL as Long-Term Prognostic Biomarkers after Heart Transplantation

**DOI:** 10.1155/2018/6243529

**Published:** 2018-08-29

**Authors:** Sonia Mirabet, Alvaro García-Osuna, Pablo Garcia de Frutos, Andreu Ferrero-Gregori, Vicens Brossa, Laura Lopez, Ruben Leta, Joan Garcia-Picart, Josep M. Padro, José Luis Sánchez-Quesada, Juan Cinca, Jordi Ordonez-Llanos, Eulalia Roig

**Affiliations:** ^1^Cardiology Department, Hospital Santa Creu i Sant Pau, CIBERCV, Barcelona, Spain; ^2^IBB-Sant Pau, Barcelona, Spain; ^3^Clinical Biochemistry Department, Hospital Santa Creu i Sant Pau, IIB-Sant Pau, Barcelona, Spain; ^4^Department of Cell Death and Proliferation, IIBB-CSIC, IDIBAPS, Barcelona, Spain; ^5^Cardiac Surgery Department, Hospital Santa Creu i Sant Pau, Barcelona, Spain

## Abstract

**Antecedents:**

Cardiac allograft vasculopathy (CAV) is a frequent complication limiting the long-term (>1 year) survival after heart transplantation (HTx). CAV is initiated by endothelial dysfunction and can lead to severe cardiovascular (CV) complications. Since CAV is often clinically silent, biomarkers could help identifying HTx patients at risk of CAV and their severe complications.

**Aim:**

Evaluate the clinical yield of high-sensitivity cardiac troponin T (hs-cTnT), marker of cardiomyocyte damage, and the soluble form of AXL (sAXL), biomarker of endothelial dysfunction, to assess the prognosis of long-term cardiovascular (CV) events occurring after HTx.

**Methods:**

96 patients were evaluated at least > 1 year after HTx. CAV was evaluated by coronary angiography or multisliced tomography, and hs-cTnT and sAXL measured 6 months before or after CAV evaluation. Patients were followed during 42 ± 15 months for a combined end point including cardiac death, angina or acute myocardial infarction, left ventricular ejection fraction < 50%, or heart failure not due to an acute rejection.

**Results:**

51 patients (53%) presented CAV at evaluation; 21 of them had CV events. Hs-cTnT (56 ± 45 versus 20 ± 18 ng/L; *p* = 0.04) and sAXL concentrations (98 ± 51 versus 26 ± 26 ng/L; *p* = 0.01) were significantly higher in patients with CV events. Hs-cTnT (HR 1.03; 95% CI 1.015–1.042, *p* = 0.0001) and sAXL (HR 1.01; 95% CI 1.001–1.019, *p* = 0.02) were independent predictors of CV events. A hs-cTnT concentration < 21 ng/L, detected by AUC ROC, predicted the absence of CV events with a predictive value of 91%; sAXL did not add more predictive value to hs-cTnT. Survival free of CV events was 92% in patients with hs-cTnT < 21 ng/L and 57% in those with hs-cTnT > 21 ng/L (*p* < 0.001).

**Conclusion:**

Hs-cTnT, but not sAXL, measured during the long-term follow-up of HTx patients appears as a helpful biomarker to identify patients at low risk of adverse CV outcomes.

## 1. Introduction

Despite the improvement of long-term (>1 year) survival after heart transplantation (HTx), several clinical conditions such as neoplasms, graft failure, infections, and cardiac allograft vasculopathy (CAV) limit it [1, 2]. CAV is the most important cause of cardiovascular (CV) adverse events during follow-up of HTx. CAV is initiated by immunologic and inflammatory phenomena causing endothelial dysfunction and damage. Endothelial damage leads to intimal growth of the coronary epi- and endocardial vessels [3]. CAV is often clinically silent and symptoms can only appear in its advanced stages as acute heart failure (HF) or sudden death. Although CAV has been associated with adverse outcomes, the progression of the disease is variable, making uncertain the prediction of the associated CV events. Some patients may experience a rapid deterioration, while others will remain stable for long time periods [4]. Efforts have been addressed to identify biomarkers predicting acute rejection; but results were disappointing [5–10]. Indeed, the role of biomarkers to predict the long-term outcomes of HTx patients is still not well known. Thus, the analysis of recently developed biomarkers linked to the mechanisms involved in CAV development could be worth for improving the risk stratification of HTx patients.

The soluble form of AXL (sAXL), a tyrosine kinase receptor, is considered a biomarker of endothelial dysfunction and associated with myocardial ischemia and heart failure [11]. Cardiac troponins, when measured with high-sensitivity methods (hs-cTn), are not only specific biomarkers of myocardial damage [12] but also sensitive indicators of minor grades of such damage [13]. Both low-grade myocardial necrosis and endothelial dysfunction typically occur in HTx patients developing CAV. The aim of the present study was to evaluate hs-cTnT and sAXL as prognostic biomarkers of long-term (>1 year) adverse outcomes after HTx.

## 2. Material and Methods

### 2.1. Study Design

**Figure 1:**
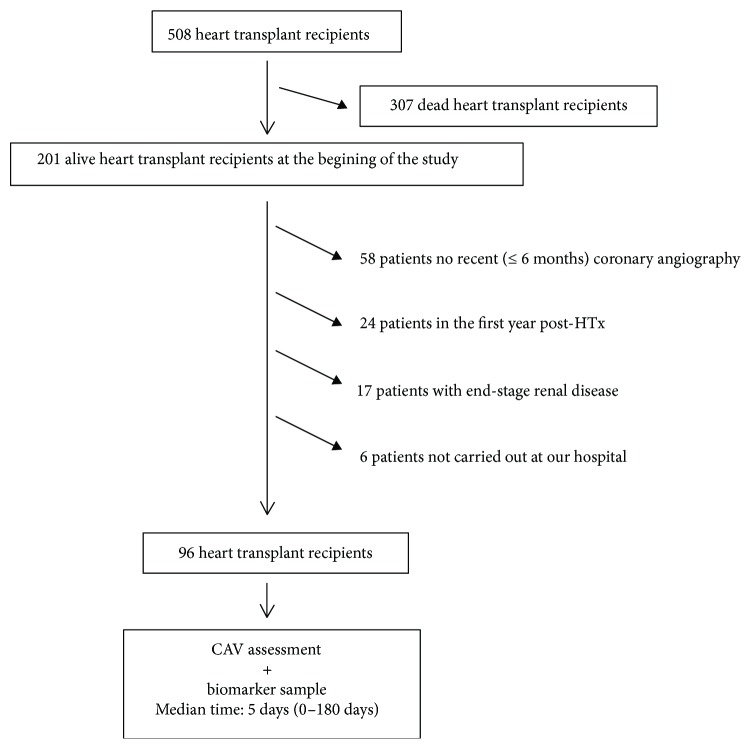
Flowchart of included patients.

Since the year 1984, our center performed 508 HTx; of them, 201 were alive at the beginning of this study and 96 fulfilled the inclusion criteria, that is, >1 year after the HTx, CAV assessment in the 6 months before/after blood was obtained for biomarker evaluation, an estimated glomerular filtration rate (eGFR) ≥30 mL/min/1.73 m^2^ and a follow-up in our center. Of the 105 excluded patients, 58 had no recent (±6 months) CAV assessment, 24 were in the first year post-HTx, 17 had eGFR < 30 mL/min/1.73 m^2^, and 6 were followed outside our hospital (Figure 1). All patients included in the study gave written informed consent to participate. The study was approved by our internal review board.

### 2.2. Clinical Variables

Donor and recipient ages at the HTx time, recipient CV risk factors, etiology (ischemic or nonischemic) of HF leading to HTx, total ischemic time during HTx, acute rejection episodes (first year after HTx), and cytomegalovirus (CMV) infection evaluated monthly during the first year post-HTx, immunosuppressive therapy, and renal function were registered. CMV infection was managed by a preemptive strategy or with prophylactic therapy. Acute rejection was assessed by endomyocardial biopsy (EMB) and was defined according to the International Society for Heart and Lung Transplantation grading system for acute cardiac allograft rejection [14]. Rejection score was defined as the ratio of EMBs with rejection grade > 2R to total EMBs during the first year of follow-up after HTx [15]. CV events were registered during the follow-up as a combined clinical endpoint including cardiac death, acute myocardial infarction or angina, left ventricular ejection fraction (LVEF) <50%, or HF not due to an acute rejection. All included patients were followed up until December 2016.

According to the HTx protocol of our center, screening for CAV was based on serial coronary angiography at 1 month, 1 year, 2 years, every 5 years, and whenever it was clinically indicated. Since the year 2012, coronary multislice computed tomography (MSCT) was also introduced after the first year post-HTx, based on its reported accuracy for CAV detection and good correlation with coronary angiography findings [16, 17]. The coronary angiograms and MSCT images were graded from 0 to 3 according to ISHLT consensus [18]. Patients were classified as non-CAV or CAV grade 1, 2, 3, that is, mild, moderate, or severe CAV, respectively.

### 2.3. Biomarkers

Biomarkers were measured on blood drawn on the same day, but previously to the procedure, or at the time of regular biochemical assessment conducted either 6 months before or after coronary angiography/MSCT. In fact, venous blood samples (lithium heparin) were obtained one month after or before coronary angiography/MSCT in more than half of the evaluated patients. Median time from blood drawing and CAV assessment was 5 days (range from 0 to 180 days). Blood was centrifuged, aliquoted, and stored frozen at −80°C until assayed. Plasma concentrations of sAXL were measured by an enzyme-linked immunosorbent assay (ELISA) previously described [19]; final concentrations were obtained from two replicates of each sample. The mean within- and between-assay imprecision (as coefficient of variation, %CV) was 6.45 and 9.21, respectively. Hs-cTnT was analyzed using an electrochemoluminometric assay in a Cobas e601 platform (Roche-Diagnostics, Basel, Switzerland). The within- and between-assay imprecision at the mean concentration observed and the instrument used was 1.2% and 2.9%, respectively, as %CV.

### 2.4. Statistical Analysis

Continuous variables are expressed as the mean ± standard deviation (SD) or as median (interquartile range) and differences by Student's *t*- or Mann-Whitney *U* tests whenever appropriate. Categorical variables are presented as frequency and percentage. Differences in the categorical variables were assessed by the *χ*^2^ test or Fisher's exact test. Hs-cTnT, sAXL, and plasma creatinine values as variables with a *p* value < 0.1 and recipient age as a clinical meaningful variable were included in the multivariate models; a backward elimination method was used to identify independent predictors of CV events. A logistic regression model was built to evaluate variables associated to CV events and the value best predicting CV events was obtained from the area under curve ROC (AUC ROC) analysis.

Factors predicting CV events were assessed by Cox multivariate regression; the model included only the main effects of the predictors, without any interaction term or treatment (due to assignment bias in an observational study design). The proportional hazard assumption was evaluated by the Schoenfeld residuals test. The discriminative ability of the model was assessed by the C-statistic. The best cut-off values were identified by AUC ROC, and Kaplan-Meier survival plot was generated for the CV events with comparison by the log-rank test.

The internal validity of the final predictive models was tested for 500 bootstrap resamples, using the “rms” package in the R Project for Statistical Computing. Missing data were imputed using the “mice” package in R (Multivariate Imputation by Chained Equations) whenever necessary (*n* = 5) [20, 21]. A two-sided *p* < 0.05 was considered statistically significant. Data were analyzed with the statistical packages SPSS 24 and R 3.2.

## 3. Results

Table 1 summarizes the main demographic and clinical characteristics of HTx subjects subdivided according to the event occurrence or absence. Mean age was 47 ± 16 years, 78% were men, and the average time from HTx was 9 ± 7 years. Not surprisingly, 79% of the patients had a plasma creatinine ≤ 133 umol/L (1.5 mg/dL), because advanced renal failure was an exclusion criterion for coronary angiography and study inclusion. There were no significant differences between patients with and without events in the recipient and donor age, total ischemia time, score rejection, and rate of CMV infection. Patients were mainly immunosuppressed with combined therapies, particularly including tacrolimus, mycophenolate mofetil, and steroids, and in some cases, cyclosporine or azathioprine; 95% of the patients were receiving statins.

Fifty-one patients (53%) presented CAV and of grade 1 in 27, grade 2 in 5, and grade 3 in 19. Patients with CAV were younger and had higher acute rejection scores and plasma creatinine levels than patients without CAV. There were no significant differences between the two groups in donor's age, total ischemic time, rate of CMV infection, or CV risk factors.

During the mean follow-up time of 42 ± 15 months, 21 patients presented CV events. All patients with CV events had CAV: 16 had CAV grade 3, 1 grade 2 and 4 grade 1. Six patients died of cardiovascular causes (4 CAV grade 3, 1 grade 2, and 1 grade 1), 6 had angina or AMI (5 CAV grade 3 and 1 grade 1), 9 had HF: 6 with LVEF ≤ 50% (all CAV grade 3) without acute rejection and the remaining 3 patients HF with LVEF > 50% (1 CAV grade 3 and 2 grade 1 with a severe restrictive pattern).

**Figure 2:**
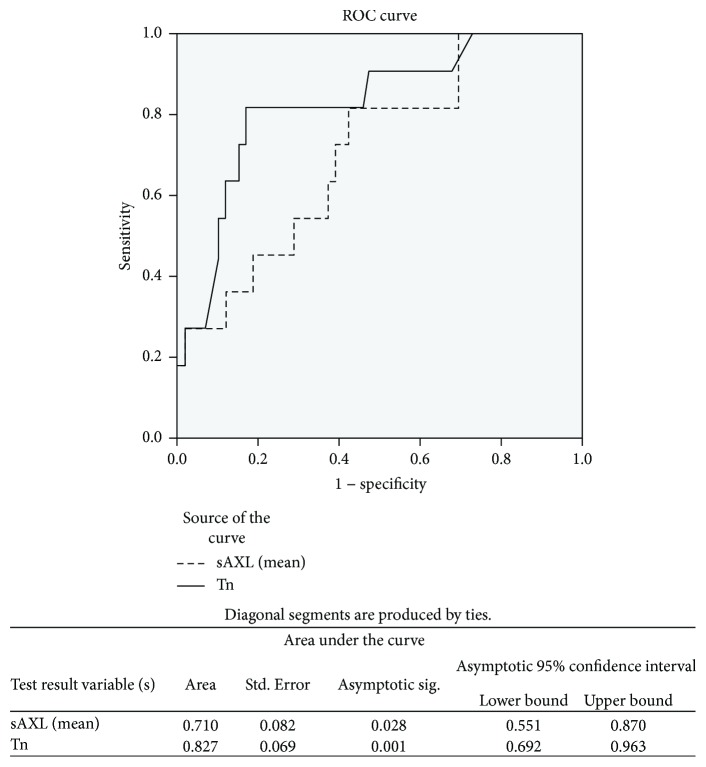
ROC curves and its *p* value for hs-cTnT and sAXL.

There was any significant correlation between biomarker concentrations and time after HTx (rho Spearman: sAXL = 0.123, *p* = 0.23; hs-cTnT = 0.168, *p* = 0.1). There existed significant differences between patients with and without CV events in hs-cTnT (56 ± 45 versus 20 ± 18 ng/L; *p* = 0.04) and sAXL concentrations (98 ± 51 versus 26 ± 26 ng/L; *p* = 0.01) (Table 1). After adjusting for clinical variables, multivariate analysis identified hs-cTnT, sAXL, and younger recipient age as independent predictors of CV events (Table 2). The addition of sAXL to hs-cTnT values and recipient age only increased the discrimination capacity to predict events by 7.3%, but the improvement was not significant *p* < 0.10. The AUC ROC analysis identified that a hs-cTnT concentration ≥ 21 ng/L had positive predictive value of CV events of only 43%, but a concentration < 21 ng/L had a negative predictive value of 91%. The best cut-off concentration for sAXL with similar negative predictive value than that of hs-cTnT was 66.5 ng/L (Figure 2). However, using this cutoff value, the positive predictive value for event prediction was lower (31%) than that of hs-cTnT.

**Figure 3:**
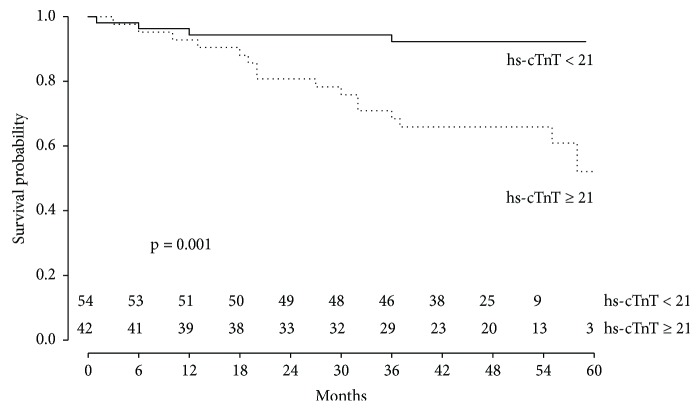
Kaplan-Meier survival curve for heart-transplanted patients according to the hs-cTnT value obtained > 1 year after transplantation and close to CAV evaluation. Comparison of survival free of cardiovascular events for recipients with hs-cTnT <21 ng/L or hs-cTnT ≥ 21 ng/L.

Survival free of CV events for a hs-cTnT < 21 ng/L was 92% compared with 57% in patients with hs-cTnT ≥ 21 ng/L (*p* < 0.001) (Figure 3).

## 4. Discussion

The current study analyzed the prognostic value of two biomarkers of cardiomyocyte lesion and endothelial dysfunction, hs-cTnT and sAXL, respectively, to predict severe CV events in long-term surviving patients (42 ± 15 months) after HTx. We found that plasma concentrations of hs-cTnT, but not those of sAXL, are strong negative predictors of the probability of suffering long-term CV events.

CAV is one of the more frequent and severe causes of CV complications in HTx patients. In the study, all patients with CV events in the follow-up have different CAV degrees, but most have the most severe form of the disease (CAV degree 3). CAV begins with an endothelial dysfunction and lesion. Consequently, increased values of sAXL, an endothelial dysfunction biomarker, should be expected in patients with CAV. We found increased sAXL concentrations in those HTx patients with CV events, but the biomarker did not add prognostic value of CV events to concentrations of hs-cTnT when analyzed in multivariable analysis.

Cardiac troponins (cTn) are specific biomarkers of cardiomyocyte injury and have become the gold standard for diagnosing myocardial lesions or infarctions [12, 13]. The new, high-sensitivity assays (Hs-cTn) allow to measure very small concentrations of cTn and distinguish between values found in healthy subjects and in subjects with subtle cardiac damage as the produced by coronary ischemia. In contrast, sAXL protein is expressed in many organs and tissues; its concentration in skeletal muscle is twice than that of cardiac tissue, and it is also expressed at higher concentrations than in heart tissue in the small bowel, colon, lungs, kidneys, and pancreas, among others [22]. Moreover, the methods developed to measure sAXL (ELISA) are not equally sensitive as the methods developed to measure hs-cTn. All these reasons can explain why circulating sAXL concentrations cannot sensitively and accurately predict the CV complications associated to CAV in our HTx population.

Small increases of hs-cTn have been associated with worse prognosis in patients with non-ST elevation acute coronary syndromes and heart failure [23]. Given that cTnT was the first cTn measurable with high-sensitivity methods (hs-cTnT), there exists much more scientific evidence for this biomarker than for others similar like hs-cTnI. Despite being accurately used in the diagnosis of myocardial lesions and infarction, hs-cTnT has been poorly studied as a marker of rejection and CV events after HTx. Routine screening and monitoring of CAV is based on serial coronary angiography or multislice computed tomography (MSCT), both exposed to morbidities related to iodine contrast infusion and X-ray exposure. Furthermore, coronary angiography is an invasive technique which cannot be performed to patients with advanced deterioration of kidney function. In a previous study, hs-cTnT concentrations at 6 weeks after HTx were independent predictors of 12-month mortality [24]. CAV can be responsible for high hs-cTnT values; but conversely, normal hs-cTnT could be predictive of CAV absence and its complications and help to identify those HTx patients at low risk of CV events to avoid an excess of coronariographies or MSCT. Recently, the high negative predictive value of low plasma hs-cTn concentrations to rule out acute HTx rejection has been described by our group and others [25, 26]. In our study, hs-cTnT concentrations < 21 ng/L showed a high negative predictive value (91%) of CV events and were associated with a 92% event-free survival in the follow-up. Based on these results, it is likely that HTx recipients with low hs-cTnT values (<21 ng/L) could undergo less frequent CAV evaluation. Recently, the AlloMap gene expression profile test has also shown a high negative predictive value to estimate the likelihood of events in patients beyond 315 days post-HTx [27]. However, this test is expensive, not widespread used, and only limited data supports its use for prognosis assessment; in contrast, hs-cTnT is an inexpensive test that can be processed in most centers and can be applied to all HTx patients regardless their renal function status.

In conclusion, based on the high negative predictive value observed in our study, the cut-off value of hs-TnT < 21 ng/L may offer a useful means to help identifying HTx patients at low risk of CV events and therefore better prognosis reducing the requirement of the more expensive and harmful image techniques.

### 4.1. Study Limitations

The relatively small size of the study combined with the fact that only 6 patients died during the follow-up precluded assessing the values of hs-cTnT to predict crude mortality.

The study included those HTx patients surviving to the intervention for >1 year and excluded the survivors with advanced renal failure. This could contribute to select those HTx survivors with a good health status. Thus, results found apply to this subgroup of HTx patients. However, this “bias selection” allows us to analyze a population on which the hs-cTnT concentration was mainly influenced by cardiac status and not by confounding factors like renal function.

Finally, although coronary angiography is the gold standard, multislice computed tomography (MSCT) has also been validated for CAV assessment [16, 17]. Since advanced renal failure was an exclusion criterion for coronary angiography, we cannot exclude advanced renal failure as an additional marker for poor survival.

## Figures and Tables

**Table 1:** Demographic and clinical characteristics and biomarkers in 96 HTx patients subdivided according to occurrence or absence of cardiovascular events.

Variables	All	No events	Events	No events versus events
	*N* = 96	*n* = 75	*n* = 21	*P*
Recipient age at HTx (years)	47 ± 16	49 ± 15	44 ± 16	0.19
Donor age (years)	40 ± 15	39 ± 13	42 ± 15	0.4
Total ischemic time (min)	183 ± 54	186 ± 52	168 ± 61	0.1
Score rejection (%)	12 ± 14	13 ± 17	12 ± 13	0.9
Cytomegalovirus infection (%)	35 (35%)	29 (39%)	6 (30%)	0.6
Plasma creatinine, umol/L	125 ± 106	104 ± 44	159 ± 120	0.002
Cardiovascular risk factors				
Pre-HTx hyperlipemia (%)	28 (29%)	18 (26%)	10 (55%)	0.02
Post-HTx hypertension (%)	53 (55%)	39 (55%)	14 (78%)	0.7
Post-HTx diabetes (%)	27 (28%)	22 (31%)	6 (33%)	0.1
CAV	51 (53%)	30 (40%)	21 (100%)	
Hs-cTnT (ng/L)	25 ± 27	20 ± 18	56 ± 45	0.04
sAXL (ng/L)	72 ± 35	26 ± 26	98 ± 51	0.01

HTx: heart transplantation; CAV: cardiac allograft vasculopathy; hs-cTnT: cardiac troponin T measured with methods of high sensitivity; sAXL: soluble form of the AXL receptor.

**Table 2:** Multivariate analysis for predictors of CV events in HTx patients.

Variables	Association with events				
	HR	95% CI	*p*	C-statistic	Corrected C-statistic
				0.855	0.836
hs-cTnT	1.03	1.015–1.042	0.0001		
sAXL	1.01	1.001–1.019	0.02		
Recipient age at HTx	0.97	0.941–0.999	<0.05		

HR: hazard ratio; CI: confidence interval; hs-cTnT: cardiac troponin T measured with high-sensitivity methods; sAXL: soluble form of the AXL receptor; HTx: heart transplantation.

## Data Availability

The data used to support the findings of this study are available from the corresponding author upon request.
